# Microgeographical structure in the major Neotropical malaria vector *Anopheles darlingi* using microsatellites and SNP markers

**DOI:** 10.1186/s13071-017-2014-y

**Published:** 2017-02-13

**Authors:** Melina Campos, Jan E. Conn, Diego Peres Alonso, Joseph M. Vinetz, Kevin J. Emerson, Paulo Eduardo Martins Ribolla

**Affiliations:** 1 Biotechnology Institute (IBTEC) & Biosciences Institute at Botucatu (IBB), Sao Paulo State University (UNESP), Sao Paulo, Brazil; 2 Department of Biomedical Sciences, School of Public Health, University at Albany (State University of New York), Albany, NY USA; 3 New York State Department of Health, Wadsworth Center, Albany, NY USA; 4 Division of Infectious Diseases, Department of Medicine, University of California, La Jolla, San Diego, CA USA; 5 Instituto de Medicina Tropical “Alexander von Humboldt,” and Departamento de Ciencias Celulares y Moleculares, Laboratorio de Investigación y Desarrollo, Universidad Peruana Cayetano Heredia, Lima, Peru; 6 Biology Department, St. Mary’s College of Maryland, St. Mary’s City, MD USA

**Keywords:** *Anopheles darlingi*, Amazonian Brazil, Malaria, Microsatellite markers, SNPs, DdRADseq

## Abstract

**Background:**

In recent decades, throughout the Amazon Basin, landscape modification contributing to profound ecological change has proceeded at an unprecedented rate. Deforestation that accompanies human activities can significantly change aspects of anopheline biology, though this may be site-specific. Such local changes in anopheline biology could have a great impact on malaria transmission. The aim of this study was to investigate population genetics of the main malaria vector in Brazil, *Anopheles darlingi*, from a microgeographical perspective.

**Methods:**

Microsatellites and ddRADseq-derived single nucleotide polymorphisms (SNPs) were used to assess levels of population genetic structuring among mosquito populations from two ecologically distinctive agricultural settlements (~60 km apart) and a population from a distant (~700 km) urban setting in the western Amazon region of Brazil.

**Results:**

Significant microgeographical population differentiation was observed among *Anopheles darlingi* populations *via* both model- and non-model-based analysis only with the SNP dataset. Microsatellites detected moderate differentiation at the greatest distances, but were unable to differentiate populations from the two agricultural settlements. Both markers showed low polymorphism levels in the most human impacted sites.

**Conclusions:**

At a microgeographical scale, signatures of genetic heterogeneity and population divergence were evident in *Anopheles darlingi*, possibly related to local environmental anthropic modification. This divergence was observed only when using high coverage SNP markers.

**Electronic supplementary material:**

The online version of this article (doi:10.1186/s13071-017-2014-y) contains supplementary material, which is available to authorized users.

## Background

The prevalence of malaria in tropical and subtropical regions [1] is due mainly to environmental conditions that are suitable for the survival of the vector anopheline mosquitoes through the extrinsic incubation period of *Plasmodium* [2]. Among Neotropical countries, Brazil has the highest proportion of malaria cases, and nearly all transmission occurs in the Amazon region [1] where *Anopheles darlingi* is the primary vector. Four main factors of *An. darlingi*’s life history have contributed to its pivotal role in *Plasmodium* transmission: susceptibility to human *Plasmodium* species; anthropophilic or opportunistic behavior [3–5]; rapid adaptability to local environmental modification [6, 7]; and the ability to blood feed successfully inside and outside houses [8, 9].

Deforestation and microclimate change that accompany human activity can significantly increase the human biting rate and other vector biology parameters in anopheline vectors across the globe [6, 7, 10, 11], though this may be site-specific [12–16]. Differences in environmental conditions have contributed to *An. darlingi* population structure spatially [6, 17, 18] and temporally [19, 20]. In Amazonian Brazil, rural settlements are subjected to geographical change by human interventions, for example, agriculture development, forest degradation and increases in house numbers. In general, the more recently occupied settlements, covered by a greater proportion of forest, have the greatest abundance of *An. darlingi* and the highest proportion of malaria cases compared with older settlements where there is increased deforestation and urbanization, and fewer malaria cases, a phenomenon described as frontier malaria [21, 22]. In the present study, we analyze *An. darlingi* populations from three endemic areas, ranging from a rural to an urban environment. Different proportions of anthropogenic (built) environment between urban and rural settings may lead to ecological segregation in breeding sites, resulting in divergence/speciation, as observed in *An. gambiae* (*s.l*.) in Cameroon [23].

Population genetic studies in the context of vector biology have used a variety of molecular markers, among them microsatellites and single nucleotide polymorphisms (SNPs). The former is a multiallelic marker that provides valuable polymorphism information, and it has been an important tool for numerous population genetics studies in *An. darlingi* and other vector species [24–26]. In *An. darlingi*, microsatellite markers have revealed moderate to high levels of genetic heterogeneity; subpopulations have been found at a macrogeographical scale (greater than 150 km apart) [17, 27, 28], and more surprisingly, seasonally related genetic subpopulations [20]. Single nucleotide polymorphisms have become popular for population genomics due to improvements in next generation sequencing and progressive cost reduction [29, 30]. Restriction-site Associated DNA sequencing (RADseq) and derivative approaches that generate SNP datasets have been used successfully to investigate genetic features in anophelines [31, 32]. For example, a recent study used SNPs to solve a long-stranding controversy about the presence or absence of a species complex in *An. darlingi* by supporting the existence of three genetic clusters (putative species) within this vector in Brazil at a large scale [33]. This study may explain some previously incongruous findings [34, 35], but does little to clarify population structure of *An. darlingi* populations at a fine geographical scale in a heterogeneous landscape such as the Amazon region. Here, we inferred genetic divergence in *An. darlingi* populations at a local scale.

## Methods

### Mosquito collections

**Fig. 1:**
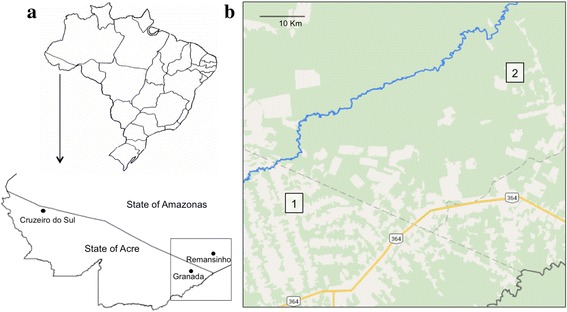
Collection region of Amazonian Brazil. **a** Map of Brazil showing Acre and Amazonas states and the three collection localities. **b** Satellite image depicting different forest degradation in Granada (1) and Remansinho (2). Settlements are connected by BR 364 highway (*yellow*) and Rio Iquiri (*blue*)

Mosquitoes were collected outdoors (peridomestic, within 10 m of each house) in two rural settlements, Granada and Remansinho in March 2012. Outdoor samples from the urban site of Cruzeiro do Sul were collected in March 2013 (Fig. 1). Collections were performed using human landing catch by the authors (MC and PERM). All specimens were morphologically identified [36] as *An. darlingi* and stored at -20 °C (Table 1).

**Table 1:** Location, number of specimens, and genetic marker used for microsatellite and ddRADseq analyses (for additional details see Additional file 3: Table S6)

Code	Location	Region	State	Latitude	Longitude	Microsatellites	ddRADseq
GRA	Granada	Acrelandia	Acre	-9.752	-67.071	59	15
REM	Remansinho	Labrea	Amazon	-9.497	-66.582	60	16
CZS	Cruzeiro do Sul	Cruzeiro do Sul	Acre	-7.625	-72.673	56	14

### Microsatellite genotyping

DNA was prepared from each mosquito with 5% Chelex solution (BioRad, Hercules, USA). Nine microsatellite loci were genotyped for 175 *An. darlingi* specimens by PCR using fluorescently labeled reverse primers (FAM, NED, or HEX; Applied Biosystems, Foster City, USA) previously described [20, 27]. Amplified fragments were separated by capillary electrophoresis in an ABI 3700 Applied Biosystems and analyzed with GeneMarker software (SoftGenetics, State College, USA). The presence of null alleles was tested in MICRO-CHECKER [37]. Estimates of expected heterozygosity (*H*_*E*_), allele richness (*Rs*), and private allele (*P*) were performed in FSTAT v 2.9.3.2 [38].

### SNP genotyping

#### Double digest restriction associated DNA sequencing (ddRADseq)

DNA from 45 individual *An. darlingi* specimens (see Table 1 for sample sizes in three locations) was extracted using ReliaPrep™ Blood gDNA kit (Promega, Madison, USA) and its concentration was estimated using a Qubit fluorometer (Invitrogen, Carlsbad, USA). The sample size for ddRADseq analysis was based on previous study with *Anopheles darlingi* in Brazil [33]. Double restriction digestion of 200 ng of high quality genomic DNA with *EcoR*I-*Msp*I restriction enzymes was performed in a 40 ul reaction volume and then purified with AMPure XP beads following the manufacturer’s protocol. A pair of customized adapters (P1 and P2) were designed including Nextera® Index Primers (Illumina, San Diego USA) complement sequence, to perform the indexing with Nextera® DNA Sample Preparation Kit (Illumina) (Additional file 1: Table S1). The working stock dilution of hybridized adapters P1 (0.3 µM) and P2 (4.8 µM) was ligated to the digested DNA (T4 DNA Ligase, Promega). After another purification with AMPure XP beads, DNA was size selected on an agarose gel to 350–400 bp and purified again. PCR amplification for Nextera® indexing was carried out to generate Illumina sequencing libraries, according to these cycling conditions: an initial denaturation step at 72 °C for 3 min and at 95 °C for 30 s, followed by 16 cycles of 95 °C for 10 s, annealing at 55 °C for 30°, elongation at 72 °C for 30 s, and a final extension cycle at 72 °C for 5 min, then each PCR product was purified one last time. Samples with distinct multiplexing indices were combined in equimolar ratios to compose a final library for sequencing. The library quantification was made with KAPA library quantification kit in a qPCR reaction. The samples were pooled, normalized and denatured, and finally loaded on the Illumina reagent cartridge. One library was paired-end sequenced in 150-cycles in a Miseq (Genetic Department Facility, Sao Paulo State University).

Stacks v1.31 [39] pipeline was used to identify SNP loci within and between individuals. Briefly, all sequence reads were quality filtered using the default parameters of stacks component *process_radtags*. Then, each individual’s sequence reads were aligned to the *An. darlingi* reference genome [40] using Bowtie2 with default parameters [41], and stacks component ref_map.pl was used to generate the genotype data (see Additional file 2: Text S1 for parameters used). Stacks was used to generate genotypes from a single SNP position (parameter - write_single_snp from stacks component *populations*) for each RAD locus, which passed through a minimum allele sequence depth of 5, as used by Emerson and collaborators [33], that was called in at least 50% of individuals, considering only one population. The last parameter certified no population bias in the SNPs selection.

### Statistical and structural analyses

A Bayesian clustering analysis with STRUCTURE [42] was performed assuming the admixture model and assuming correlated allele frequencies among populations. We conducted 20–40 independent runs for each *K* value (ranging from 1–4) using a 100,000 ‘burn-in’ period and 1,000,000 generations. The optimal value of *K* was inferred using the Evanno method [43] implemented in structureHarvester [44]. Locus-specific and pairwise *F*_*ST*_ estimates of genetic diversity, as well as Hardy-Weinberg (HW) equilibrium tests and linkage disequilibrium (LD) between pairs of microsatellite loci were computed using ARLEQUIN 3.5 [45]. The nominal significance level was *α* = 0.05; when multiple tests were performed, the sequential Bonferroni procedure was applied. In addition, as microsatellites are known to give precise, but often downwardly biased estimates of genetic differentiation [46], we include estimates of corrected Hendrick *G*_*ST*_ (*G"*_*ST*_) [47], using GenoDive package [48], that standardize the differentiation estimate relative to the maximium differentiation possible for the level of homozygosity observed. Adegenet package [49] in R software [50] was used to perform principal components analysis (PCA) and discriminant analysis of principal components (DAPC).

## Results

### Genetic diversity and structure of microsatellite data in *An. darlingi*

**Fig. 2:**
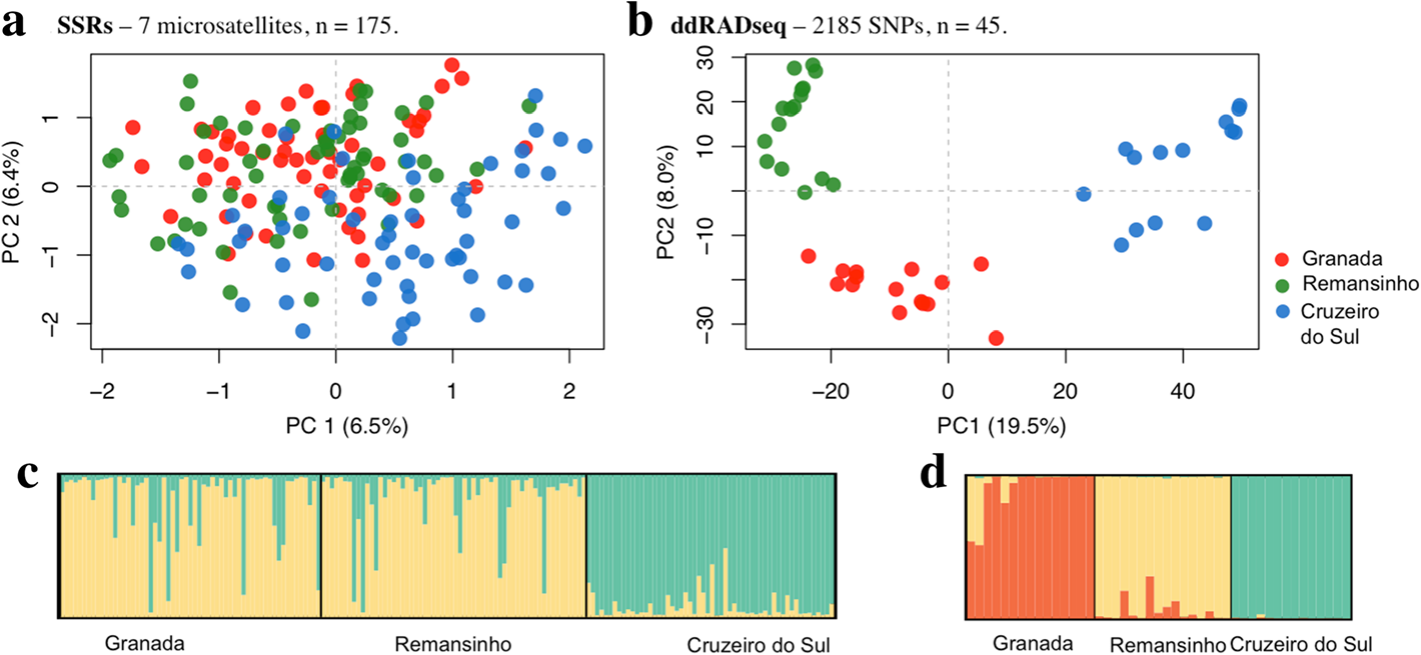
STRUCTURE and Principal Components Analysis (PCA) of individual *An. darlingi* genotypes from the three localities. **a** PCA using microsatellites dataset. **b** PCA using SNPs dataset. **a**, **b** Colors reflect population assignment: Granada, *red*; Remansinho, *green* and Cruzeiro do Sul, *blue*. In parentheses along x and y-axes: percent variance explained by PC1 and PC2. **c** STRUCTURE results from analysis of microsatellites loci variation (*K* = 2). **d** STRUCTURE results from SNP variation (*K* = 3). **c**, **d** Each column represents an individual and colors reflect genetic clusters assignment (cluster 1, *light yellow*; cluster 2, *light green*; cluster 3, *orange*)

**Fig. 3:**
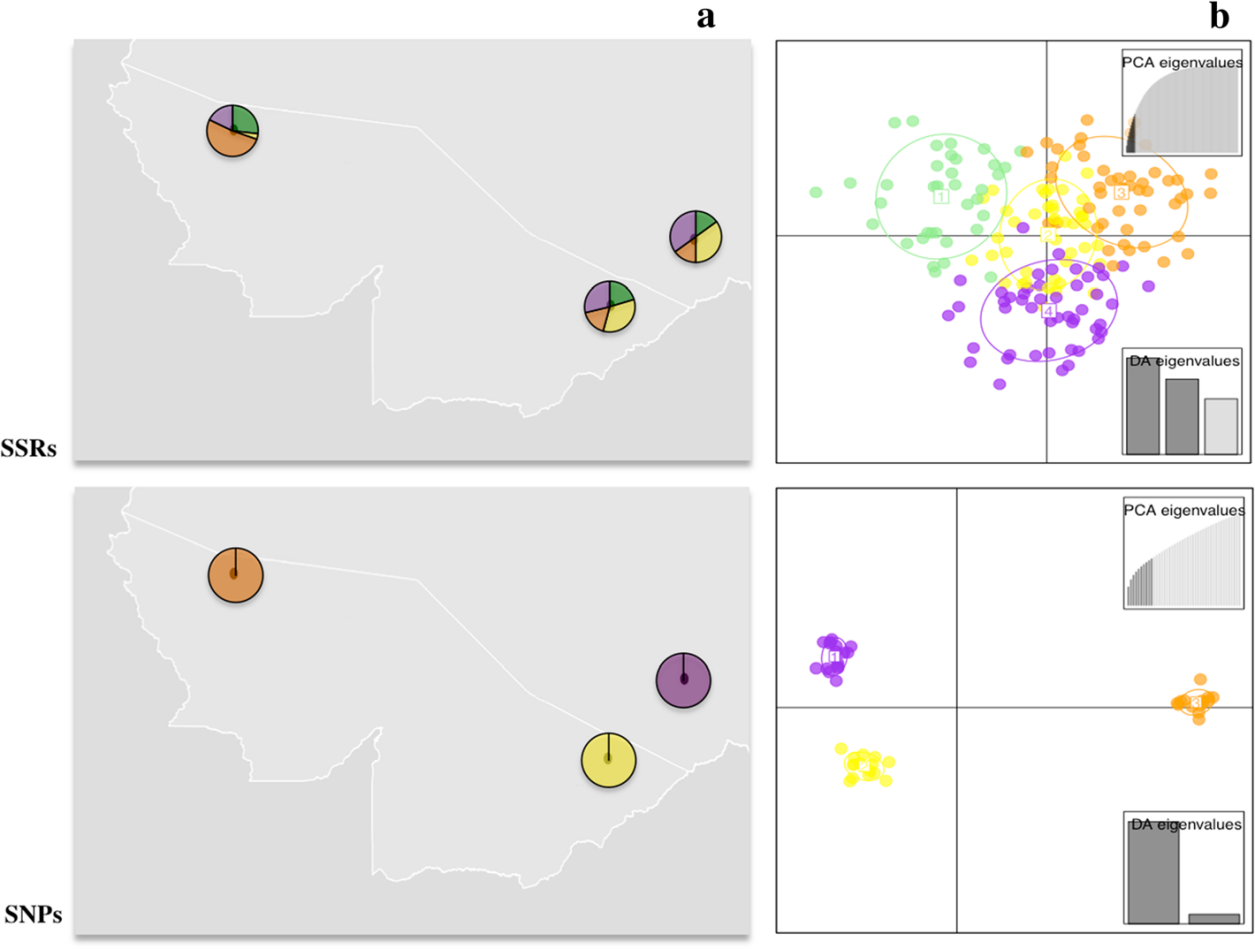
Discriminant analysis of principal components (DAPC) of individual *An. darlingi* genotypes from the three localities. **a** Pie charts of the cluster assignment distribution in Granada, Remansinho and Cruzeiro do Sul plotted in a map. **b** Ordination of the clusters in two axes. Colors represent genetic clusters (*light green*, *yellow*, *orange*, *purple*)

One hundred and seventy-five specimens from rural settlements Granada and Remansinho, and urban Cruzeiro do Sul in western Amazonian Brazil were genotyped using nine microsatellite markers that were polymorphic in all groups analyzed. Estimates of *H*_*E*_, *F*_*is*_ and allelic richness (*Rs*) per locality and sampling period are presented in Additional file 1: Table S2. The number of alleles per locus present within a population ranged from 4 to 43. Significant departures from Hardy-Weinberg equilibrium were detected at loci ADC29 and ADC138 in all samples (Additional file 1: Table S2) and these markers were excluded in population structure analysis. The highest values of allelic richness (*Rs*) and number of private alleles (*P*) were observed in Remansinho (Additional files 1: Tables S2, S3). Grouping the rural settlements of Remansinho and Granada resulted in 46 private alleles (Additional file 1: Table S3). Estimates of *F*_*ST*_ were significant only between Cruzeiro do Sul and each of the two rural settlements Granada and Remansinho (Table 2). In the locus-by-locus analysis, *F*_*ST*_ ranged from 0.019 (*P* < 0.0001) to 0.133 (*P* < 0.0001) (Additional file 1: Table S4). STRUCTURE analysis of microsatellite alleles revealed two genetic clusters consisting of Cruzeiro do Sul and Granada + Remansinho (Fig. 2c); nonetheless PCA did not clearly separate the three locations (Fig. 2a). DAPC showed evidence of four genetic clusters, with all clusters represented in all three geographical locations (Fig. 3a, b).

**Table 2:** Pairwise *F*_*ST*_ values from microsatellite and ddRADseq data of *An. darlingi* populations. Lower left values are from microsatellites while upper right values are from ddRADseq data

	Granada	Remansinho	Cruzeiro do Sul
Granada		0.072*	0.181*
Remansinho	0.002		0.118*
Cruzeiro do Sul	0.043*	0.042*	

*P < 0.001

### Genetic diversity and structure of ddRADseq data in *An. darlingi*

From 54,616,244 ddRAD tag sequences (NCBI SRA BioProject PRJNA298241), around 46 million sequences passed several levels of quality filtering in the process_radtags program (Stacks v. 1.31 [39], details in Additional file 3: Table S6), and 33.9% (± 2.06 SD) of this set of reads was aligned to the *An. darlingi* genome [40]. An average of 17,401 (± 6,248 SD) ddRAD loci were genotyped per sample. After filtering, 2185 SNPs were found in at least 50% of all 45 individuals. Pairwise *F*_*ST*_ values were significant between Cruzeiro do Sul and both rural settlements, as well as between settlements (Table 2). Remansinho had the highest number of private alleles and polymorphic sites (Additional file 1: Table S5).

STRUCTURE analysis of SNP variation revealed three genetic clusters (*K* = 3), which were assigned to each collection point (Fig. 2d). PCA separated the three populations based on SNP variation (Fig. 2b). DAPC partitioned the genetic variation into three genetic clusters, where each contains a unique collection point (Fig. 3).

Overall diversity was also calculated using *G"*_*ST*_ index. The results also showed a higher level of diversity with SNPs *G"*_*ST*_ = 0.138, 0.121–0.155 (2.5–97.5% CI) than with microsatellites *G"*_*ST*_ = 0.119, 0.065–0.219 (2.5–97.5% CI), but the values were not so different as in *F*_*ST*_ estimates.

## Discussion

The observed population genetic divergence among collection localities was higher with SNPs than with microsatellites markers in both model-based analysis using Bayesian Analysis (BA) with STRUCTURE, *F*_ST_ estimation, and non-model-based analysis by DAPC. BA assumes a model-clustering method based on allele frequencies at each locus, and probabilistically each individual is assigned to a number of genetically distinct clusters (*K*) [42]. In the present study, BA revealed two clusters by microsatellites; one essentially characterized the specimens from urban Cruzeiro do Sul and the other categorized specimens from both rural settlements (Fig. 2c). However, the optimal number of clusters based on BA analysis of SNPs was three, and each cluster defined only one location (Fig. 2d). It is also worth noting that the admixture between clusters was lower in the SNP analysis, highlighting the discrimination among genetic clusters. Both marker types showed higher *F*_*ST*_ estimates between Cruzeiro do Sul and the two rural settlements than between the two rural settlements, however the estimates based on SNPs were more than 4-fold higher than those based on microsatellites between Cruzeiro do Sul and rural settlements, and 35-fold higher between Granada and Remansinho (Table 2).

High-throughput methods using next generation sequencing that analyze a subsample of the genome, such as ddRADseq, have two major advantages compared to microsatellites, the need of smaller sample sizes and also no need of prior knowledge of the genomic sequence [30]. In the present study, the number of SNPs generated and used was much higher than the number of microsatellites, which could contribute to increased statistical power in the analysis. Nevertheless, other studies have shown the efficiency of SNP genotyping even when a small number of SNPs are used [51–53]. SNP analyses have corroborated microsatellite-based findings, and have presented superior accuracy, robustness and recovered finer population structure when compared to microsatellite analysis [53, 54].

PCA of the SNP data separates individuals originating in Cruzeiro do Sul from those in Granada and Remansinho (separated by ~ 700 km) along the first principal component that explains 19.5% of the variation (Fig. 2b). Nonetheless, at a finer scale (~60 km apart), individuals from Granada and those from Remansinho were also separated along principal component 2, which accounted for 8% of the total variation. This was validated by DAPC analysis, which found three distinct clusters, uniquely identifying individuals to their appropriate geographical population. No clear separation of the populations was reflected in the PCA for the microsatellite data (Fig. 2a), and DAPC revealed four distinct genetic clusters, equally partitioned among the three geographical locations.

Rural settlements are in constant flux due to human interventions such as agricultural development, forest degradation, and increased and often mobile human populations [22, 55]. Such anthropogenic environmental changes along with seasonal climate variation, such as temperature, rainfall and humidity, influence the survival, density and distribution of mosquitoes [16, 20]. The highest risk of malaria transmission in such settlements, common in Amazonian Brazil and Peru [7, 22, 56], is typically in the newest settlements where migrants have no previous exposure to *Plasmodium* and little effective shelter from bites of infected *An. darlingi*. Proximity of residences to potential mosquito breeding-sites is also associated with the likelihood of becoming infected with *Plasmodium* [57–59]. The two rural settlements of the present study, Granada and Remansinho, have experienced anthropogenic landscape modification to different degrees because of their relative ages; Granada was initiated in 1982 and Remansinho 25 years later [55, 60]. Regardless of the genetic marker used (microsatellites or SNPs) the two rural settlements samples presented higher genetic diversity than the sample from the urban area. Even between the rural settlements, genetic diversity was highest in mosquitoes from the newer settlement, Remansinho, which has a greater proportion of intact forest compared to the older settlement of Granada [60]. Our findings in *An. darlingi* support the hypothesis that deforestation may be associated with a loss of genetic diversity [61, 62]. Deforestation enhanced survivorship, reproductive fitness and increased population growth potential of *An. gambiae* in the western Kenyan highlands [63, 64]. A similar scenario may be occurring in *An. darlingi* in settlements that are at different temporal points in the frontier malaria model. Once roads have been built for settlements, deforestation to clear space for housing and crop planting is a priority.

Small-area interventions may be an effective approach for malaria control and elimination in the neotropics and globally, once transmission pockets have been identified and characterized [65–67]. Each locality has peculiar environmental characteristics and thus, it might have different anopheline population genetic backgrounds, which may lead to differences in vector capacity and competitiveness. For an intervention to be successful, it is essential to be able to precisely identify genetic differences between vector populations and subpopulations at a microgeographical scale.

## Conclusion

In this study, we provide evidence that the detection of microgeographical population structure at a fine scale is only robust when we apply high-resolution molecular typing techniques, since conventional approaches based on microsatellite markers may underestimate overall genetic distances in closely related vector populations. In our view the application of ddRADtag sequencing for genetic analysis of mosquito populations represents a suitable molecular tool to further elucidate vector population dynamics in malaria endemic areas*.*

## Additional files

Additional file 1: Table S1. Double digest RADseq primer and adapters sequences for *An. darlingi.* **Table S2.** Estimates of *Rs*, *H*_*E*_ and *F*_*IS*_ of *An. darlingi* microsatellite loci in three Brazilian populations. **Table S3.** Estimates of private alleles in *An. darlingi* using microsatellite loci. **Table S4.** Locus-by-locus analysis of *An. darlingi* microsatellite loci. **Table S5.** Summary of ddRADseq dataset containing all positions (variant and fixed) from the three *An. darlingi* populations. (DOCX 27 kb)
Additional file 2: Text S1. Bash script with commands used to run the Stacks pipeline and STRUCTURE analysis. (TXT 4 kb)
Additional file 3: Table S6. Per-individual *An. darlingi* detail of the number of sequence reads and unique stacks genotyped. (XLSX 18 kb)

## Abbreviations

BA: Bayesian analysis
DAPC: Discriminant analysis of principal components
ddRADseq: double digest restriction associated DNA sequencing
LD: Linkage disequilibrium
PCA: Principal components analysis
SNP: Single nucleotide polymorphism
