# Supplementary material for: Microgeographical structure in the major Neotropical malaria vector Anopheles darlingi using microsatellites and SNP markers
Source: Parasit Vectors. 2017 Feb 13;10:76. doi: 10.1186/s13071-017-2014-y (PMC5307779; doi:10.1186/s13071-017-2014-y)
Supplement: Additional file 1: Table S1. — Double digest RADseq primer and adapters sequences for An. darlingi. Table S2. Estimates of Rs, H E and F IS of An. darlingi microsatellite loci in three Brazilian populations. Table S3. Estimates of private alleles in An. darlingi using microsatellite loci. Table S4. Locus-by-locus analysis of An. darlingi microsatellite loci. Table S5. Summary of ddRADseq dataset containing all positions (variant and fixed) from the three An. darlingi populations. (DOCX 27 kb) [file 13071_2017_2014_MOESM1_ESM.docx]

**Additional file 1**

**Table S1.** Double digest RADseq primer and adapters sequences for *An. darlingi*

| Name |  | Sequence |
| --- | --- | --- |
| PCR primer | P5 | 5' AATGATACGGCGACCACCGAGATCTACAC[i5]**TCGTCGGCAGCGTC** 3' |
| Nextera® Index Kit | P7 | 3' **GGCTCGGGTGCTCTG**[i7]TAGAGCATACGGCAGAAGACGAAC 5' |
| P1 adapter | 1.1 | 5´ **TCGTCGGCAGCGTC**AGATGTGTATAAGAGACAG 3' |
| (EcoRI site) | 1.2 | 3' AGCAGCCGTCGCAGTCTACACATATTCTCTGTCTTAA 5' |
| P2 adapter | 2.1 | 5´CGCTGTCTCTTATACGTCCTCTT 3' |
| (HpaII site) | 2.2 | 3' GACAGAGAATATGTGTAGA**GGCTCGGGTGCTCTG** 5´ |

[i7] and [i5] are the Illumina barcode sequences.

**Table S2.** Estimates of *Rs*, *H_E_* and *F_IS_* of *An. darlingi* microsatellite loci in

three Brazilian populations

| **Site name** |  | **Locus** | | | | | | | | | |
| --- | --- | --- | --- | --- | --- | --- | --- | --- | --- | --- | --- |
| **N** |  | **ADSP2** | **ADMP9** | **ADC28** | **ADC29** | **ADC138** | **ADC02** | **ADC137** | **ADC110** | **ADC01** | **All Loci** |
| Granada | *Rs* | 2.987 | 10.890 | 8.767 | 16.623 | 9.999 | 15.505 | 15.299 | 15.753 | 30.017 | 13.982 |
| 59 | *A* | 3 | 11 | 9 | 17 | 10 | 16 | 16 | 16 | 31 | 14 |
|  | *H_e_* | 0.329* | 0.808 | 0.823 | **0.895*** | **0.863*** | 0.83 | 0.869 | 0.912 | 0.953 | 0.809 |
|  | *F_IS_* | 0.394 | 0.027 | 0.069 | 0.181 | 0.432 | 0.056 | -0.017 | 0.032 | 0.005 | 0.153 |
| Remansinho | *Rs* | 3.882 | 10.719 | 10.754 | 19.381 | 12.926 | 17.494 | 14.520 | 15.479 | 28.123 | 14.809 |
| 60 | *A* | 4 | 11 | 11 | 20 | 13 | 18 | 15 | 16 | 29 | 15 |
|  | *H_e_* | 0.253 | 0.753 | 0.841 | **0.843*** | **0.899*** | 0.857 | 0.859 | 0.875* | 0.951 | 0.792 |
|  | *F_IS_* | -0.121 | 0.022 | 0.109 | 0.347 | 0.416 | -0.032 | 0.069 | 0.11 | 0.003 | 0.123 |
| Cruzeiro do Sul | *Rs* | 3.000 | 5.997 | 8.942 | 9.981 | 7.998 | 9.000 | 12.000 | 8.997 | 25.512 | 10.159 |
| 56 | *A* | 3 | 6 | 9 | 10 | 8 | 9 | 12 | 9 | 26 | 10 |
|  | *H_e_* | 0.232 | 0.718 | 0.699 | **0.784*** | **0.575*** | 0.812 | 0.863* | 0.81 | 0.922 | 0.713 |
|  | *F_IS_* | 0.042 | 0.006 | 0.056 | 0.412 | 0.433 | 0.099 | 0.258 | 0.053 | 0.012 | 0.111 |
| All sites | Rs | 3.303 | 9.670 | 9.561 | 18.567 | 11.924 | 17.046 | 14.302 | 14.852 | 30.504 | 14.414 |
|  | A | 4 | 11 | 11 | 23 | 13 | 22 | 17 | 17 | 43 | 18 |

N= sample size; Rs= Allelic Richness per locus; population based on minimum sample size of 56 individuals; H_e_= expected heterozygosity; *F_IS_*= fixation indices; in bold: significant P-value after Bonferroni correction; *presence of null alleles

detected by Micro-checker [20,27].

**Table S3.** Estimates of private alleles in *An. darlingi*

using microsatellite loci

|  | Number of private alleles |
| --- | --- |
| Granada | 8 |
| Remansinho | 15 |
| Cruzeiro do Sul | 8 |
| Granada + Remansinho | 46 |

Among populations (*p*=0.575); Granada + Remansinho,

(*p*=0.002)

**Table S4**. Locus-by-locus analysis of

*An. darlingi* microsatellite loci

|  | Fixation Index | |
| --- | --- | --- |
| *Locus* | *F_ST_* | *P*-value |
| ADSP2 | 0.02990 | 0.00684 |
| ADMP9 | 0.02747 | 0.00000 |
| ADC28 | 0.04538 | 0.00000 |
| ADC29 | 0.02652 | 0.00098 |
| ADC138 | 0.13340 | 0.00000 |
| ADC02 | 0.02136 | 0.00098 |
| ADC137 | 0.03106 | 0.00000 |
| ADC110 | 0.02960 | 0.00000 |
| ADC01 | 0.01902 | 0.00000 |

| **Table S5.** Summary of ddRADseq data set containing all positions (variant and  fixed) from the three *An. darlingi* populations | | | | | | | |
| --- | --- | --- | --- | --- | --- | --- | --- |
| Location | Number of individuals | Private | Sites | Variant Sites | Polymorphic  Sites | % Polymorphic Loci |  |
| Granada | 15 | 278 | 330835 | 2185 | 1050 | 0.3174 |  |
| Remansinho | 16 | 627 | 330684 | 2184 | 1523 | 0.4606 |  |
| Cruzeiro do Sul | 14 | 339 | 286847 | 1897 | 885 | 0.3085 |  |
